# Evaluating Meridian-Sinew Release Therapy for the Treatment of Knee Osteoarthritis

**DOI:** 10.1155/2013/182528

**Published:** 2013-06-19

**Authors:** Song Wei, Zhi-Huang Chen, Wei-Feng Sun, Geng-Peng Zhang, Xiao-Hao Li, Chun-Fu Hou, Liu-Dan Lu, Lu Zhang

**Affiliations:** ^1^Department of Traditional Chinese Medicine, Guangzhou General Hospital Of Guangzhou Military Command, Guangzhou 510010, China; ^2^Guangzhou University of Traditional Chinese Medicine, Guangzhou 510405, China

## Abstract

*Objective*. In recent years, public health experts have concluded that the impact of osteoarthritis is equal in magnitude to that of cardiovascular disease. Osteoarthritis of the knee is prevalent in the elderly population; however, there are currently no effective treatments for this condition. In this study, we investigated the efficacy of “meridian-sinew release,” a newly developed technique which entails using a meridian-sinew scope and a meridian-sinew knife to treat osteoarthritis of the knee. *Methods*. Patients (*N* = 90) with knee osteoarthritis were prospectively randomized to meridian-sinew release therapy, acupuncture therapy, or drug therapy groups, respectively. Outcome evaluation included pain, stiffness, physiological function, total symptom score, and overall changes in the condition. *Results*. After 12 weeks, patients' general assessment (GA) and doctors' general assessment (GA) of the condition were not significantly different among the three groups. However, significant differences in primary endpoint pain, joint stiffness, and total symptom score were found between the meridian-sinew group and the acupuncture group and between the meridian-sinew group and the control group (*P* < 0.05). No adverse events occurred during the trial. *Conclusion*. Our study suggests that meridian-sinew release therapy can improve knee osteoarthritis, alleviate joint pain, and improve functional movement disorder. It is a safe and effective treatment for knee osteoarthritis.

## 1. Introduction

 Osteoarthritis is a degenerative joint disease that occurs mainly in the elderly. It is characterized by the peripheral (i.e. a, osteophytes) erosion of articular cartilage, bone hypertrophy, and subchondral sclerosis. Osteoarthritis is the most common form of arthritis in the elderly and also one of the main causes of disability in that population [1, 2]. Due to the fact that the knee joint is a peripheral axial, weight-bearing joint, it is most commonly affected by osteoarthritis [3]. Studies have shown that the incidence of knee osteoarthritis in people over age 65 is 60%–70%, with the incidence rate reaching up to 85% in the population over age 75 [4]. In the United States, approximately 21 million Americans suffer from the disease [5], and it is estimated that the total cost of treating arthritis may be close to 2.5% of its gross domestic product (GDP). Therefore, the impact of osteoarthritis on public health has recently been compared to the impact of cardiovascular disease [6]. The main clinical symptoms of knee osteoarthritis are pain and joint stiffness [7]. However, no effective treatment for knee osteoarthritis has been developed to date. Usual care, based on the guidelines published by the American College of Rheumatology (ACR) and the European Association of Rheumatology Union, focuses on alleviating the symptoms of pain and stiffness and maintaining or improving physical function [8, 9]. Our goal in this study was to find a more effective treatment that would reduce joint pain and disability and prevent and mitigate cartilage degradation [10]. Conventional treatment for knee osteoarthritis is designed to control symptoms and pain and includes nonsteroidal anti-inflammatory drugs, glucosamine, topical analgesics, intra-articular injection of sodium hyaluronate and surgical treatment [11, 12]. However, none of these treatments are considered curative and are often accompanied by side effects ranging from patient discomfort to liver and kidney damage. Most knee osteoarthritis patients are not satisfied with the recurring side effects of conventional drug therapy [13, 14]. As a result, many knee osteoarthritis patients use complementary and alternative therapies. In China, Traditional Chinese medicine (including acupuncture) has been used for thousands of years and has been shown to be particularly efficacious for treating pain, especially when related to joint diseases such as osteoarthritis [15–18]. Due to the limitations and side effects of conventional therapies, more and more people are turning to complementary and alternative medicine. Therefore, it is necessary to explore and scientifically evaluate the efficacy of new therapies for this disorder.

In meridian-sinew release therapy, a physician uses a “meridian-sinew scope” to observe local tissue while simultaneously using a “meridian-sinew knife” to loosen and release adhesions. Indications include joint and myofascial pain, as well as the local, refractory pain and inflammation in rheumatoid disorders. This may be a new, effective treatment for these disorders that can replace conventional therapy. In China, this therapy has been used to treat a variety of rheumatoid disorders involving joint swelling and pain, including knee osteoarthritis and rheumatoid arthritis [19–22]. In our hospital, we have found that this therapy can relieve the pain and stiffness associated with knee osteoarthritis, with results that can be maintained for a relatively long period of time. We also believe it can slow down the degeneration of articular cartilage and maintain and improve joint function [21, 22]. The technique causes minimal injury to local tissue without compromising the overall structure of the knee. There are minimal bleeding and rapid patient recovery. Our results are consistent with research that has shown that soft tissue release can effectively alleviate myofascial pain in the forearm caused by external humeral epicondylitis [23].

We performed a randomized, controlled study in order to further evaluate the efficacy and safety of meridian-sinew release therapy for treating knee osteoarthritis. The basic design of this study was to quantify and compare the efficacy of meridian-sinew release therapy, acupuncture, and routine drug treatment for knee osteoarthritis. We hoped that our data would help guide policy makers in determining whether this therapy should be more made more widely available as a new, safe, and effective treatment for osteoarthritis of the knee.

## 2. Materials and Methods

### 2.1. Subjects

Patients hospitalized in Guangzhou General Hospital Of Guangzhou Military Command from January 2008 to December 2011 were recruited. The diagnosis of knee osteoarthritis was made according to the Kellgren grading standard, and patients with grade II or III were included; this was also consistent with the American College of Rheumatology standards [24–26]. We applied the following criteria for inclusion in the study: (i) age 45 years or older; (ii) diagnosis of osteoarthritis of the knee of at least 6 months duration; (iii) moderate to severe pain during most days throughout the past months and use of analgesics for at least 1 month; (iv) willing and able to complete the study protocol. The exclusion criteria were intra-articular corticosteroid injection into the knees within 4 weeks preceding the study and severe, unstable chronic illness (including but not limited to congestive heart failure, chronic renal failure, tumors in the knee, autoimmune diseases such as rheumatoid arthritis, ankylosing spondylitis, congenital deformity of the knee, and trauma-induced osteoarthritis of the knee). During the study period, patients were treated with conventional drug therapy (glucosamine sulfate capsules: take 2 capsules 3 times daily, manufacturer: Rottapharm Srl, Italy, approval number: X19990394; celecoxib: take 1 capsule daily, manufacturer: Pfizer Pharmaceuticals LLC, USA, approval number: J20080059) but were not allowed to begin new drug treatment or change the dosage of current medication. The study was approved by the Ethics Committee of Guangzhou General Hospital Of Guangzhou Military Command Area, and all patients signed informed consent forms.

Estimation of sample size was based on the results of previous studies and then calculated according to the calculation formula for sample size estimation, with a clinical efficacy increase of 25% from the original level. The calculation formula was as follows: *n* = (*U*_*α*_ + *U*_*β*_)^2^2*P*(1 − *P*)/(*P*_1_ − *P*_0_)^2^ [27].

### 2.2. Interventions and Randomization

Patients were told that the study had been designed to evaluate and compare the efficacy of meridian-sinew release therapy, acupuncture and conventional drug therapy for knee osteoarthritis and that they would be required to give up other forms of treatment for the duration of the study. Patients were randomly assigned to the meridian-sinew release therapy group, acupuncture group or control group through computer-generated random numbers. Patients in the meridian-sinew release therapy group were treated with conventional drug therapy plus meridian-sinew Release therapy, patients in the acupuncture group were treated with conventional drug therapy plus acupuncture, and patients in the control group were treated with conventional drug therapy without any other additional treatment.

### 2.3. Treatments in Detail

#### 2.3.1. Meridian-Sinew Release Group

**Figure 1:**
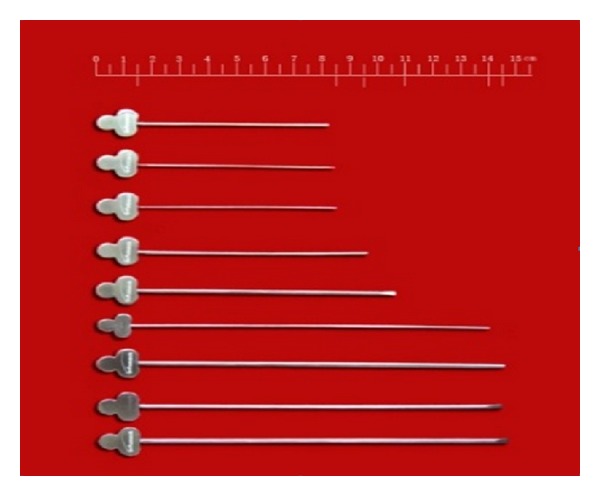
Meridian-sinew knives.

**Figure 2:**
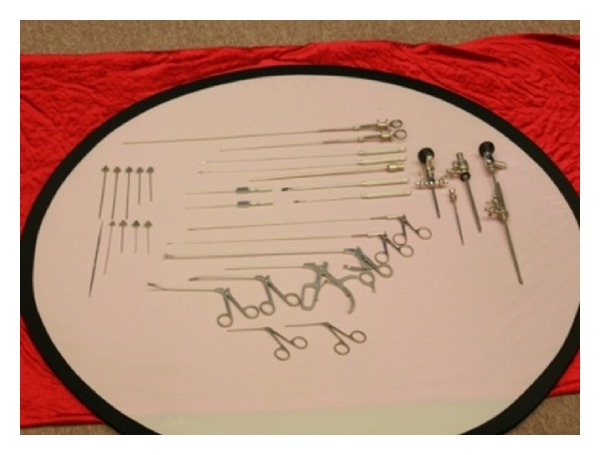
Meridian-sinew scope and surgical instuments.

A minimally invasive technique has been invented (Figures 1 and 2) to release connective tissue adhesions and alleviate joint and myofascial pain [19–22]. The meridian-sinew scope and meridian-sinew knife technology are based on the concept and description of the “Nine Needles” found in the Han dynasty classic of traditional Chinese medicine, *The Yellow Emperor's Inner Canon*. This modality is an improvement on the ancient method and is now used in modern-day China to treat rheumatoid pain. The course of treatment for meridian-sinew release therapy treatment was 4 weeks. In the 1st week, the meridian-sinew scope was used to release adhesions. The procedure was as follows: local anesthesia was given according to the conventional standard, and a small incision was made with a scapel in order to insert the meridian-sinew scope into the anterolateral aspect of the knee joint. The meridian-sinew scope was inserted into the incision, and anatomical changes in the structure of intra-articular tissue were observed, and the scope was slowly moved toward the lesions. In addition to the forward movement, side movement was also performed to order to inspect the suprapatellar bursa, patellar joint space, medial tibial space, medial meniscus, medial crypts, fossa intercondyloidea, lateral tibial clearance, meniscus lateralis, and cornucopia in the joint cavity. The needle knife was inserted to release adhesive tissue in the articulatory antrum. At the same time, the joint cavity was flushed with water and drained through a standard drainage tube until the fluid became clear. 

In the 2nd, 3rd, and 4th weeks, patients were treated with the meridian-sinew knife only. Treatment sites were chosen based on the textbook, *China Meridian Sinews* [28], and consisted of major sites (similar to acupuncture or acupressure points) along the pathways of meridian sinews in the vicinity of the knee. The main sites included *binxia* (the lower edge of the patella, patella articular surface), *binwai* (the outer edge of the midpoint of patella), *binneixia* (the lower margin of patella, the initial part of the medial patellar retinaculum vice), *chengshanci* (triceps surae fascia and tendon junction), *chengshanwai* (lateral gastrocnemius muscle belly and hamstring nodes), *weiyangci* (the lateral end of popliteal transverse line, unit two quadriceps medial margin), *ciliaoci* (the inner side of the femoral condyle), the head of fibula (the upper edge of fibula), the medial tibial condyle (tibia epicondyle anterior medial eminence), and *xiguanci *(the medial part of the medial condyle of the tibia, medial margin). We marked points with gentian violet, injected local anesthesia (lidocaine 0.2%, 1 mL), and inserted the meridian-sinew knife until it reached the surface of the bone. We released the adhesions with horizontal movements and opened the meridian-sinew pathways with vertical movements. This was performed 3 times a week for 3 weeks.

#### 2.3.2. Acupuncture Group

In the acupuncture group, at least 6 acupoints were chosen for treatment from the following: ST34, ST35, ST36, SP9, SP10, BL40, KI10, GB33, GB34, and LR8. In addition, at least 2 distal points were chosen from the following: SP4, SP5, SP6, ST6, BL20, BL57, BL58, BL60, BL62, and KI3 [29]. Acupuncture was performed using needles 40 mm in length with a diameter of 0.25 mm (Huatuo, Suzhou Medical Instruments Factory, Suzhou, China). Needles were inserted perpendicularly with the aid of a guide tube and moved to a depth of 10 mm using slight rotation and thrusting. Deqi sensation was obtained and reported by the participants as a dull ache, numbness, or heaviness. Needle manipulation was repeated approximately every 5 min to maintain the Deqi sensation, and each treatment session lasted for 30 min. Treatment was given 3 times a week for 4 weeks.

#### 2.3.3. Control Group

Patients were given conventional drug therapy.

### 2.4. Outcome Evaluations

The knee with the worst arthritis pain (target joint) at screening was the joint used for evaluation of efficacy. The main outcome indicator was the visual analogue scale (VAS) for pain (the Western Ontario and McMaster University Osteoarthritis Index visual analogue scale (WOMAC) version 3.1), western Ontario province and the University of McMasters Osteoarthritis Index pain (VAS WOMAC version 3.1), including pain (five questions), stiffness (two questions), physical function (17 questions), and total symptoms (24 questions) [30, 31]. WOMAC evaluation, with scores from 0 to 100 mm (0 represents no pain and 100 represents the most severe pain), was implemented before the treatment and after 12 weeks of treatment.

The patient general assessment, physician general assessment, and the MOS item short from health survey (SF-36) (version 2), which are secondary endpoints, were used to assess the overall health-related quality of life and were collected before the start of treatment and after 12 weeks of treatment. The patient general assessment and physician general assessment were scored on a five-point Likert scale for overall arthritis disease status (0 = very well, 1 = well, 2 = moderate, 3 = poor, and 4 = very poor) and response to therapy (0 = excellent response, 1 = good response, 2 = moderate response, 3 = slight response, and 4 = no response). SF-36 was chosen due to its previous application in a variety of diseases including osteoarthritis efficacy studies [32–34].

### 2.5. Statistical Analysis

An intent to treat-based analysis was performed using the SPSS 16.0 system. The changes from baseline to week 12 between treatment and placebo groups were considered significant for independent samples *t*-test *P* values <0.05, (95% confidence level).

## 3. Results

**Figure 3:**
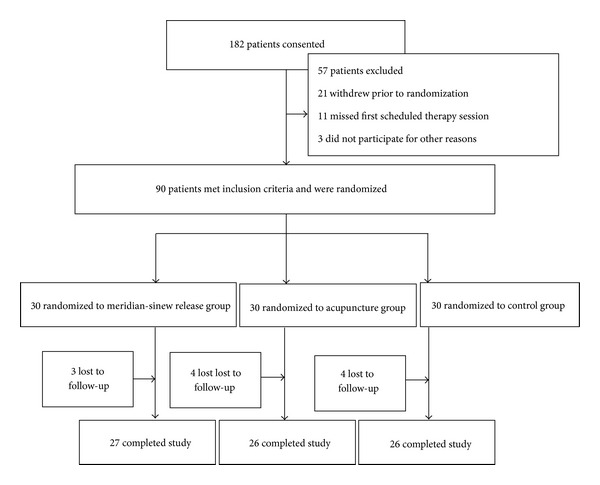
Flow chart of the distribution of the study cohort.

**Figure 4:**
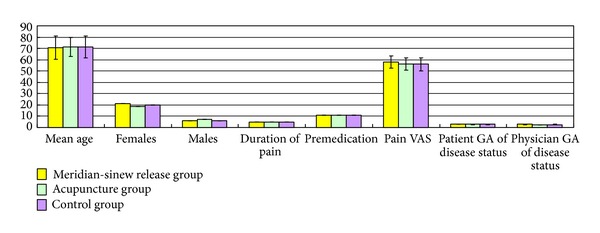
Baseline comparison of the randomized groups by treatment types.

Between January 2008 and December 2011, we identified 182 patients with a diagnosis of knee osteoarthritis. We excluded 92 patients because they did not meet the inclusion criteria, were not willing to participate in the study, or did not in strictly adhere to the study regimen. We enrolled 90 patients with knee osteoarthritis in the study.  Figure 3 shows the patient recruitment, allocation, follow-up losses, and the exclusion.  Figure 4 describes the baseline characteristics of the patients. 3 patients in the meridian-sinew theory group dropped out, citing pain levels, surgery, or personal reasons. 4 patients in the acupuncture group dropped out, two due to pain levels and two for personal reasons. 4 patients in the control group dropped out, one due to pain, one due to surgery, and two due to personal reasons.

**Figure 5:**
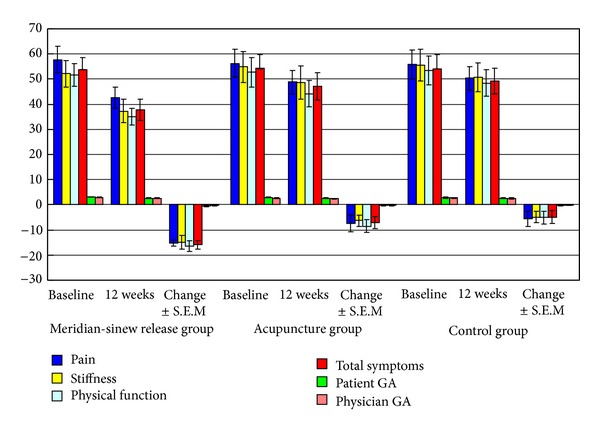
WOMAC, patient and physician GAs.

The results of WOMAC are shown in Figure 5. In the meridian-sinew release group, changes in the primary endpoint of pain at week 12 were significantly greater than in the acupuncture group or the control group (*P* = 0.041 and *P* = 0.028, resp.) (*P* < 0.05). Changes in physiological function in the meridian-sinew release group were also significantly better at week 12 than the acupuncture group or the control group (*P* = 0.045 and *P* = 0.030, resp.) (*P* < 0.05). Improvement in joint stiffness was greater in the meridian-sinew release group compared to the acupuncture group after 12 weeks of treatment and the control group (*P* = 0.048 and *P* = 0.032, resp.) (*P* < 0.05). Total symptom score changes in the meridian-sinew release group were also higher than those in the acupuncture group and the control group (*P* = 0.046 and *P* = 0.031, resp.) (*P* < 0.05). Changes in the patients' general assessment and physicians' general assessment were not significant in any of the three groups at week 12 (*P* > 0.05, Figure 5). However, the patients' general assessment and physicians' general assessment in the meridian-sinew release group and the acupuncture group had a significant trend towards improvement. There were no significant differences between the patient's general assessment and the physician's general assessment. The results of the SF-36 nine domains of quality of life survey showed that at week 12 the only significant change was in the physical status domain in the meridian-sinew release group, with a mean change of 17.12 (SD = 21.05, *P* = 0.023) (*P* < 0.05). In the acupuncture group, a mean change of 12.69 (SD = 23.81, *P* = 0.175) (*P* > 0.05) was observed in the physical status domain, and in the control group, the physical status domain at 12 weeks showed a mean change of 11.71 (SD = 24.08, *P* = 0.192) (*P* > 0.05). No notable change was found in the other eight domains (*P* > 0.05). There were no adverse events during the trial.

## 4. Discussion

These results suggest that meridian-sinew release therapy is a safe and effective method for the treatment of knee osteoarthritis. It was shown to be more effective than either routine acupuncture or routine drug therapy for the alleviation of pain and improvement of physiological function. In a recent systematic review, acupuncture was shown to relieve the pain of chronic knee osteoarthritis and improve movement function, both in the short-term (2–15 weeks) and long-term (26–52 weeks) tests [35]. In addition, the results of another clinical trial also indicated that acupuncture can relieve knee pain and improve function scores of knee osteoarthritis [36].

In our study, significant differences in primary endpoint pain, joint stiffness, and total symptom score were found between the meridian-sinew release group and acupuncture group and between the meridian-sinew release group and control group. This suggests that meridian-sinew release therapy can significantly improve knee osteoarthritis pain, joint stiffness, and physical function. The changes of overall disease status of arthritis in patients' general assessment and doctors' general assessment did not change significantly in any of the three groups. We suspect that the treatment time was too short or the sample size was too small to assess overall changes in the disease state; therefore, our next step is to explore this question with a larger and longer study design. In the meridian-sinew release group, the arthritis disease status had obviously improved after treatment. This suggests that the meridian-sinew therapy may be able to improve the overall disease status of knee osteoarthritis given a longer course of treatment. During the study, the meridian-sinew release group patients had no adverse events, indicating that meridian-sinew release therapy is safe.

This study used a randomized, controlled clinical trial design. However, because meridian-sinew release therapy and acupuncture are quite different methods, it was impossible to blind the patients. Instead, we adopted three separate principles: a single-blinded operator, a blinded observer, and statistical separation.

 Knee osteoarthritis is an arthropathy characterized by hyperostosis due to degeneration of cartilage in the knee. Pain is the most typical clinical manifestation and the primary treatment target of the disease [37]. In the theory of traditional Chinese medicine, knee osteoarthritis is classified as a meridian-sinew disease. Meridian-sinews, also known as the 12 meridian-sinews, are a body system that is formed when *Qi* from the twelve meridians is distributed in the muscles, tendons, ligaments, and joints. The 12 meridian-sinews are dependent upon the 12 regular meridians. The pathway and distribution of the meridian-sinews are basically the same as those of the twelve meridians, with the significant exception that they all move towards the heart. The distribution of the twelve meridian-sinews has 4 features: “knot” (*jie*), “gather” (*ju*), “scatter” (*san*), and “distribute” (*bu*). Meridian-sinews “knot” or “gather” in the joints, which means that they converge mostly in the wrist, elbow, shoulder, neck, ankle, knee, hip, and other joints. They are said to “scatter” or “distribute” in the chest, back or head, and face. Although some meridian-sinews are broadly distributed throughout the body cavity, they do not directly connect with the viscera. The functions of the meridian-sinew are to constrain bones, control the flexion-extension of joints, and to maintain the body's ability to have normal, physiological movement. Dr. Xue believes that the meridian-sinews serve as an essential link between various tissues and organs of the human body, and, relative to the regular meridians, they have a more tangible structure and more easily quantified functions [38]. In the view of modern medicine, meridian-sinews are basically equivalent to connective tissue in terms of function. Anatomically, they include muscles, tendons, fascia, ligaments, joint capsules, synovial fluid, and other systems [39, 40]. The structure of the knee is composed of muscles, tendons, ligaments, joint capsule, and synovial; therefore, in traditional medicine it is said that “the knee is the confluence of tendons.” Many meridian-sinews gather and conjoin around the knee; therefore, lesions associated with the knee are related to dysfunctions of the meridian-sinew system. In fact, lesions often correspond exactly to the “knots” or “gatherings” along the course of the meridian-sinews [41].

“Meridian-sinew disease” is defined as acute and chronic disease of the muscles, tendons, and joint synovium [42]. Traditional Chinese medicine theory states that “When Wind, Cold and Dampness invade the space between the surface and the “divisions in the flesh,” (fascia-muscles) the damage leads to the formation of “foam.” When foam meets cold it coagulates, and the resultant gatherings displace and break-up the fascia-muscles, thereby causing pain.” Exogenous, pathogenic *Qi* in the form of wind, cold, and heat, excess emotions that harm the *Qi* and blood, trauma to the sinews, and cumulative fatigue may all cause body fluids to coagulate and form “foam.” The “foam” leads to swelling and pain in body. If the pathologies affecting the meridian-sinew are not resolved in a timely manner, “foam” will coagulate and change to “phlegm” [43]. Phlegm-fluid retention obstructs the meridians and leads to pathological changes like spasm, cramps, pain and stiffness. Phlegm and blood stasis entangled in the fascia' muscle may form “strips” and “nodules” along the course of the meridian-sinews course, which will obstruct the channels and eventually lead to both regional and overall (in the entire meridian system) fascia contracture.

**Figure 6:**
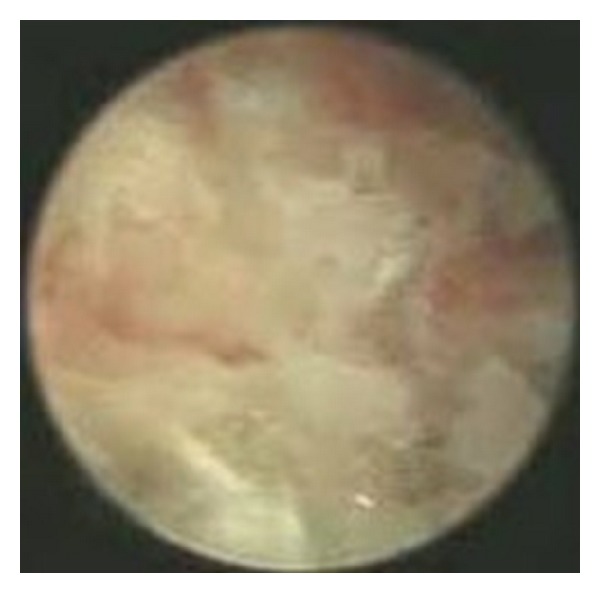
Body fluid penetrating into the cavity and creating foam.

**Figure 7:**
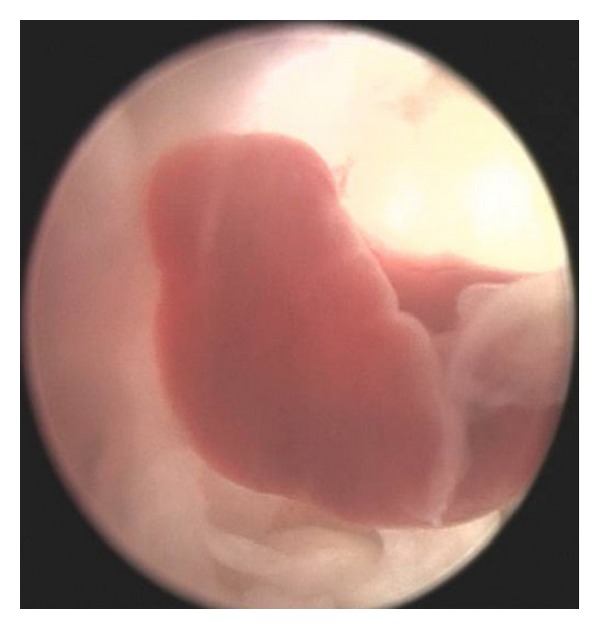
Phlegm and blood stasis obstructing collaterals.

**Figure 8:**
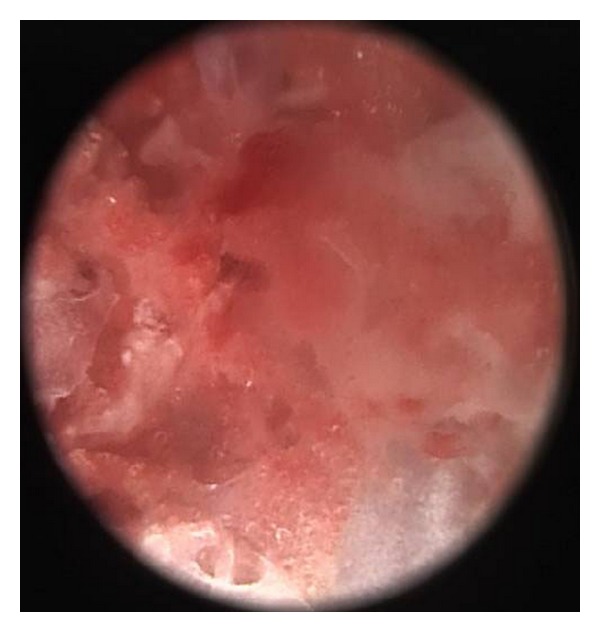
Coagulated phlegm, blood stasis, and foam.

**Figure 9:**
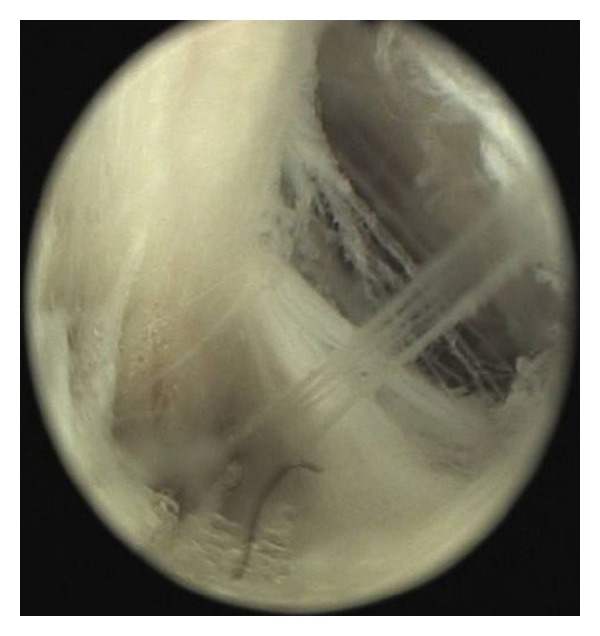
Fibroplasia.

**Figure 10:**
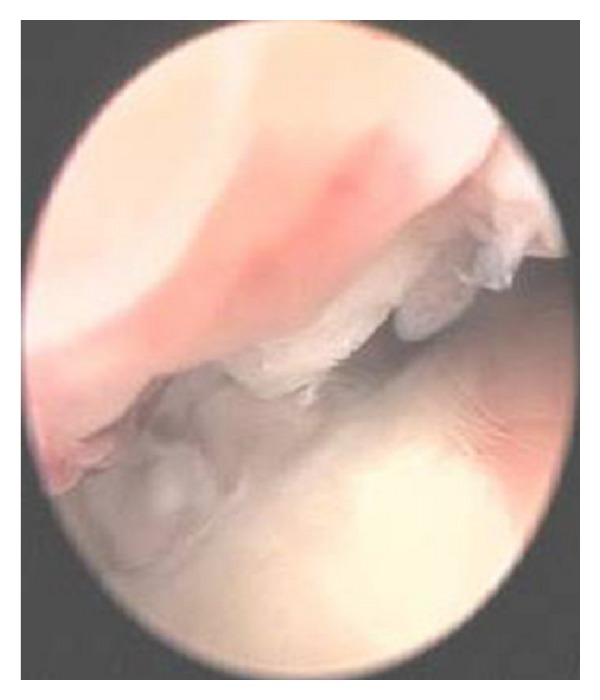
Fascial thickening.

**Figure 11:**
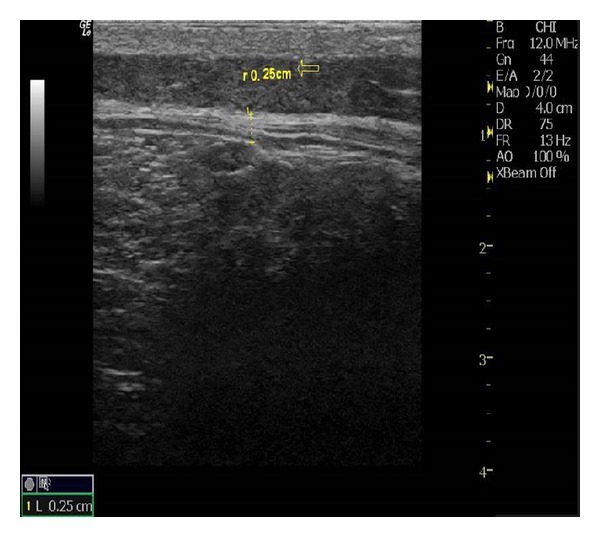
Fascia thickening under color doppler ultrasound.

In this study, the meridian-sinew scope allowed us to view various kinds of phenomena including blood stasis, foam, and phlegm (Figures 6, 7, 8, 9, and 10). Using color ultrasound, we discovered that the myofascia was significantly thickened (Figure 11). We concluded that “strips” and “nodules” hinder the movement of *Qi* and blood in meridian channels, which leads to irreversible inflammatory exudation and foam accumulation. Zhang et al. discovered a low hydraulic resistance channel along meridians through which interstitial fluid is easy to flow [44]. The channel exists within fascia meridians and can be influenced by the state of the fascia meridians. Whenever strips and nodules are formed, they may hinder the flow of interstitial fluid and cause an accumulation of inflammatory substances in the interstice, inducing pain or hyperalgesia.

Opening the fascia meridians is the key to clearing the pathways of the regular meridians. According to the theory of the meridian-sinews, this therapy can be used to release the adhesions along the fascia meridians in order to get rid of obstructions in the meridians and to decrease inflammatory exudation. This is why meridian-sinew release therapy is effective for treating the pain of arthritis.

In order to “disentangle” different structures of the body that seem to play a role in disease [45], Mr. Wei invented the meridian-sinew scope and meridian-sinew knife, drawing inspiration from the “large needles” and “long needles” described in the Nine Needles and Twelve Sources chapter of the Yellow Emperor's Inner Classic. Recent clinical reports have shown that treatment with meridian-sinew scope and meridian-sinew knife has obtained satisfactory effects [19–22]. Through the meridian-sinew release treatment, adhesions in the soft tissue around the knee are released, decreasing spasms in the surrounding ligaments and tendons, effectively improving the joint gap, and thereby improving joint functions [46]. Meridian-sinew release therapy is also a mechanical process, and its mechanism of action may enhance local tissue functions and lymphatic circulation to speed up and improve metabolism in the diseased tissue. It may also enlarge interstitial fluid channels, enhancing the interstitial flow and reducing the fluid pressure, prompting the absorption of diseased tissue and substances. These lesions of absorption lead to local swelling, which further accelerates the interstitial-lymphatic circulation, thus speeding up the recovery from the disease [47].

The results from this study indicate that, compared with the acupuncture group and drug therapy group, patients in the meridian-sinew release therapy group showed more significant changes in the primary ending point pain, physiological function, anchylosis, and the total symptom score in the 12th week. The major limitation of this study was the small sample size because it was just a pilot case study. A controlled study with a larger sample size will be done in the near future.

## 5. Conclusion

This preliminary study shows that meridian-sinew release therapy should be considered as a treatment for knee osteoarthritis. It is better than acupuncture and oral medication therapy. This therapy was shown to relieve joint pain and improve function without any adverse events during the entire study. It is a safe and effective treatment for knee osteoarthritis.

## Abbreviations

GDP: Gross domestic product
ACR: American College of Rheumatology
VAS: Visual Analogue Scale
WOMAC: The Western Ontario and McMaster University Osteoarthritis Index visual analogue scale
SF-36: The MOS item short from health survey.
